# Drip-and-Ship for Thrombectomy Treatment in Patients With Acute Ischemic Stroke Leads to Inferior Clinical Outcomes in a Stroke Network Covering Vast Rural Areas Compared to Direct Admission to a Comprehensive Stroke Center

**DOI:** 10.3389/fneur.2021.743151

**Published:** 2021-11-01

**Authors:** Christian A. Taschner, Alexandra Trinks, Jürgen Bardutzky, Jochen Brich, Ralph Hartmann, Horst Urbach, Wolf-Dirk Niesen

**Affiliations:** ^1^Department of Neuroradiology, Freiburg University Medical Center, University of Freiburg, Freiburg, Germany; ^2^Faculty of Medicine, University of Freiburg, Freiburg, Germany; ^3^Department of Neurology and Neuroscience, Freiburg University Medical Center, Freiburg, Germany

**Keywords:** stroke, mechanical thrombectomy, stroke outcome, drip-and-ship, mothership

## Abstract

**Introduction:** Organizing regional stroke care considering thrombolysis as well as mechanical thrombectomy (MTE) remains challenging in light of a wide range of regional population distribution. To compare outcomes of patients in a stroke network covering vast rural areas in southwestern Germany who underwent MTE via direct admission to a single comprehensive stroke center [CSC; mothership (MS)] with those of patients transferred from primary stroke centers [PSCs; drip-and-ship (DS)], we undertook this analysis of consecutive stroke patients with MTE.

**Materials and Methods:** Patients who underwent MTE at the CSC between January 2013 and December 2016 were included in the analysis. The primary outcome measure was 90-day functional independence [modified Rankin score (mRS) 0–2]. Secondary outcome measures included time from stroke onset to recanalization/end of MTE, angiographic outcomes, and mortality rates.

**Results:** Three hundred and thirty-two consecutive patients were included (MS 222 and DS 110). Median age was 74 in both arms of the study, and there was no significant difference in baseline National Institutes of Health Stroke Scale scores (median MS 15 vs. 16 DS). Intravenous (IV) thrombolysis (IVT) rates differed significantly (55% MS vs. 70% DS, *p* = 0.008). Time from stroke onset to recanalization/end of MTE was 112 min shorter in the MS group (median 230 vs. 342 min, *p* < 0.001). Successful recanalization [thrombolysis in cerebral infarction (TICI) 2b-3] was achieved in 72% of patients in the MS group and 73% in the DS group. There was a significant difference in 90-day functional independence (37% MS vs. 24% DS, *p* = 0.017), whereas no significant differences were observed for mortality rates at 90 days (MS 22% vs. DS 17%, *p* = 0.306).

**Discussion:** Our data suggest that patients who had an acute ischemic stroke admitted directly to a CSC may have better 90-day outcomes than those transferred secondarily for thrombectomy from a PSC.

## Introduction

According to the HERMES meta-analysis that combined the data of five randomized controlled trials (RCT), mechanical thrombectomy (MTE) is of benefit to most patients with acute ischemic stroke caused by occlusion of the proximal anterior circulation [large vessel occlusion (LVO)], irrespective of patient characteristics or geographical location (1). Organizing regional stroke care in light of this new evidence has been and still is a major challenge. The current version of the American Heart Association (AHA) guidelines for the “Early Management of Patients With Acute Ischemic Stroke” clearly states that regional systems of stroke care should be developed (2). They should include (a) health care facilities that provide initial emergency care, including administration of intravenous (IV) thrombolysis (IVT) and (b) centers performing endovascular stroke treatment with comprehensive periprocedural care to which rapid transport can be arranged when appropriate. This statement has both the highest level of evidence (Level A) and the highest class of recommendation (Class I). According to the same guideline, when several IVT-capable hospital options exist within a defined geographic region, the benefit of bypassing the closest to bring the patient to one that offers a higher level of stroke care, including MTE, is uncertain (2). These statements can be read as a recommendation for the drip-and-ship (DS) model—patients are transported to the nearest primary stroke center (PSC) to have rapid diagnostic imaging and administration of IVT followed by transport to the nearest PSC for MTE, as opposed to the mothership (MS) model, where patients are directly brought to the comprehensive stroke center (CSC) to minimize time to MTE. A recent RCT which compared MTE with or without IVT in patients with acute ischemic stroke due to LVO concluded that MTE alone was non-inferior with regard to functional outcome [modified Rankin score (mRS) ≤ 2 at 90 days] within a 20% margin of confidence to MTE preceded by IVT administered within 4.5 h after symptom onset (3). Nevertheless, data on non-inferiority of MTE alone are inconclusive; thus, the question of whether patients with LVO eligible for MTE should be transported to CSC directly is still unanswered.

To address this question, we analyzed functional outcomes of patients treated for acute ischemic strokes within our regional stroke network, comparing patients treated with a DS approach to patients sent directly to our CSC for MTE.

## Materials and Methods

The data that support the findings of this study are available from the corresponding author upon reasonable request.

### Study Design

We conducted an investigator-initiated, retrospective, open-label trial involving patients with acute ischemic stroke who either were directly sent to our CSC or were secondarily transferred from a PSC to undergo MTE at the CSC between January 1, 2013, and December 31, 2016. The study protocol was approved by our Ethics Committee (Faculty of Medicine, University of Freiburg, 077/09). Members of the trial steering committee and the local investigators designed the study, collected and analyzed the data, wrote the manuscript, and made the decision to submit the manuscript for publication.

### Organization of Stroke Care

At the time of patient inclusion, our Interdisciplinary NeuroVascular Network South-West (INVAS) stroke network comprised 12 PSCs and one CSC at the University Hospital Freiburg. The catchment area of the INVAS stroke network covers a surface area of 9,357 km^2^ and a population of 2.3 million. It includes the rather densely populated Rhine valley as well as remote rural and mountainous regions of the southern black forest.^1^ Depending on the location of the patient presenting with symptoms of an acute ischemic stroke, transfer occurred either to one of the PSC or directly to the CSC. The decision of transportation destination was performed by an emergency medical team. Patients from the PSC were discussed via a telestroke platform with the attending stroke neurologist at the CSC. Patients from the PSC deemed eligible for MTE were secondarily transferred to the CSC. For definitions of PSC and CSC, please refer to the recent publication of Ernst et al. (4).

### Patients

Patients from both PSC and CSC were eligible for MTE if they were >18 years of age, had a score of 6 or higher on the National Institutes of Health Stroke Scale (NIHSS) or a relevant functional deficit, a suspected (dense vessel sign) or proven [computed tomography angiography (CTA)/magnetic resonance angiography (MRA)] LVO of the anterior circulation, or a small core infarct with an Alberta Stroke Program Early CT Score (ASPECTS) >4. Initiation of MTE had to be possible within 6 h after stroke onset. Patients from PSC had repeated magnetic resonance imaging (MRI) or CT imaging if time from initial imaging to arrival at the CSC exceeded 2 h. Patients from both the PSC and CSC who did not present contraindication to systemic lysis received a weight-adapted dose of rtPA within 4.5 h after onset of acute ischemic stroke.

### Endovascular Treatment

The majority of patients were treated under general anesthesia. The standard procedure included a 9F femoral access and 8F or 9F balloon guide catheters for the carotid artery. Thrombus retrieval stent retrievers were used for the large majority of cases (Table 1). Medical management and acute poststroke care after the procedure were consistent with the European Stroke Organization (ESO)/European Society for Minimally Invasive Neurological Therapy (ESMINT) and AHA guidelines (2, 5). A CT scan was performed within 24 h after the revascularization procedure to detect the presence of intracerebral hemorrhage (ICH).

**Table 1:** Patient demographics.

	**Mothership**	**Drip and ship**	**Drip and ship**	***p*-value**
		**total**	**received MTE**	
**Patients**	*N* = 222	*N* = 163	*N* = 110	
Age in years—median (IQR)	74 (64–82)	75 (61–80)	74.5 (62–80)	0.272
Women, *n* (%)	114 (51%)	75 (46%)	51 (46%)	0.460
NIHSS—median (IQR)	15 (12.5–18.5)	16 (11–19)	15.5 (11.3–19)	0.958
ASPECTS—median (IQR)	8 (7–10)	9 (7–9)	9 (7–9)	n.a.
IVT, *n* (%)	122 (55%)	107 (66%)	77 (70%)	0.008
**Target vessels**				0.002
ICA, *n* (%)	47 (21%)		19 (17%)	
MCA, *n* (%)	134 (60%)		51 (46%)	
Tandem occlusion, *n* (%)	41 (19%)		40 (36%)	
**Thrombectomy**				
Stent retriever, *n* (%)	220 (99%)		109 (99%)	0.852
Proximal balloon catheter, *n* (%)	204 (92%)		101 (92%)	0.223
Number of passes, median (range)	1 (0–6)		1 (0–5)	0.479
TICI 2b/3, *n* (%)	159 (72%)		80 (73%)	0.833
**Time intervals**				
Onset to first imaging (min), median (IQR)	89 (67–116)	74 (48–103)	76 (51–104)	***0.002***
Onset to IVT (min), median (IQR)	120 (89–155)	101 (86–125)	102 (87–125)	***0.002***
Onset to first DSA run (min), median (IQR)	180 (152–218)		294 (98–397)	***<0.001***
Onset to recanalization (min), median (IQR)	230 (193–275)		342 (299–405)	***<0.001***

*Drip and ship-total, sent to the comprehensive stroke center for mechanical thrombectomy (MTE); drip and ship-received MT, sent to the comprehensive stroke center and received MTE; NIHSS, National Institutes of Health Stroke Scale; IQR, interquartile range; ASPECTS, Alberta stroke program early CT score; IVT, intravenous thrombolysis; DSA, digital subtraction angiography; p-value, mothership vs. drip-and-ship-received MT. p-values that are significant are bold*.

### Clinical and Radiological Assessments

All patients underwent clinical examination at baseline and discharge. Baseline data collected from the patients' medical chart included gender, age, stroke onset time, baseline NIHSS, time of initial CT/MRI, IVT given, time of IVT, time of CT/MRI at CSC for patients transferred from PSC, time of first angiographic run, time of recanalization, 24-h NIHSS, and NIHSS at discharge.

In addition, an independent rater who did not participate in the endovascular stroke treatment of included patients, with >10 years of experience in neuroimaging (R.H.), qualitatively evaluated pre-therapeutic MR, CT, and digital subtraction angiography (DSA) images as well as final angiograms after MTE and follow-up CT at 24 h. The extent of the initial core infarct was determined on pre-therapeutic CT or diffusion-weighted imaging (DWI) using ASPECTS (6). Revascularization was assessed by applying the modified thrombolysis in cerebral infarction (TICI) classification (7). Intracranial hemorrhage was defined on follow-up CT at 24 h as symptomatic in case of parenchymal hematoma with a clinical worsening of at least four points on the NIHSS scale according to the European Cooperative Acute Stroke Study (ECASS) definition (8). In addition, subarachnoid hemorrhages were reported.

### Study Endpoints

The primary study outcome measure was disability at 90 days, as assessed by applying the mRS. Secondary clinical efficacy outcomes were NIHSS at discharge, mortality at 90 days, and the rate of symptomatic ICH (sICH), asymptomatic lobar hematoma, and subarachnoid hemorrhages. The last recorded NIHSS score was used as discharge NIHSS in patients who had deceased during hospitalization. The technical efficacy outcomes were successful reperfusion defined as angiographic demonstration of TICI scores of 2b (50–99% reperfusion) or 3 (complete reperfusion). In addition, we analyzed different time intervals including onset to first imaging, onset to IVT, onset to first angiographic run, and onset to recanalization/end of MTE for MS and DS patients. In DS patients, we additionally determined the transfer time defined as the time interval between first imaging at PSC and patient arrival at the CSC.

### Ethics

The studies involving human participants were reviewed and approved by the Ethics Committee of the Faculty of Medicine, University of Freiburg, Freiburg, Germany.

### Statistical Analysis

The data are expressed with standard descriptive statistics, including median, interquartile range (IQR) or range for ordinal and numerical variables, and absolute and relative frequencies for nominal variables as appropriate. Relative frequencies were rounded to whole numbers according to the common rules.

Comparisons among groups on baselines variables, peri-procedural time intervals, and outcome variables were made using Pearson's chi-square test for nominal variables (age, gender, IVT, target vessels, stent retrievers and catheters, mRS 0–2, TICI 2b/3, mortality, and hemorrhage data) and Mann–Whitney *U*-test for ordinal and numerical variables (age, NIHSS, ASPECTS, number of passes, and time intervals). Statistical significance was set at *p* < 0.05 for all contrasts of hypothesis. For small sample sizes with *n* < 30 (sICH and asymptomatic lobar hemorrhage), Fisher's exact test was used. Primary endpoint analysis was amended by logistic regression analysis.

The analyses were performed with IBM SPSS Statistics version 24. Outcome analyses of cerebral hemorrhages were conducted with R Studio software version 1.3.959. The figure was drawn with the use of Microsoft Excel 2013. No missing values were imputed. Statistical consulting was provided by the Institute of Medical Biometry and Statistics of the University of Freiburg.

## Results

### Baseline Results

From January 1, 2013, to December 31, 2016, 385 patients were included in the analysis population. Two-hundred and twenty-two patients were directly admitted to the CSC with acute ischemic stroke and were treated with MTE (MS). One-hundred and sixty-three patients were admitted at PSC and transferred after telestroke consultation for MTE to the CSC (DS). Of 163 patients from the DS group (DS-total), 110 patients actually were treated by MTE (DS-received MTE) at the CSC. Patient demographics, NIHSS at presentation, initial ASPECTS score, and target vessel for MTE are summarized in Table 1. Supplementary Table 1 outlines the different reasons for not performing MTE in 53 patients from the DS-total group.

Mean age was 74 years (IQR 64–82) in the MS group compared to 75 years (IQR 62–80) in the DS-total group. In the MS group, 51% (114/222) of patients treated were female, whereas 46% (75/163) of the DS-total group were women. The median NIHSS did not differ significantly between groups and was 15 (IQR 12.5–18.5) for the MS group and 16 (IQR 11–19) for the DS-total group. With median ASPECTS scores of 8 (IQR 7–10), these were slightly lower in the MS group when compared to the DS-total group with 9 (IQR 7–9). Distribution of target vessels was slightly different between groups with 60% (134/222) middle cerebral artery (MCA) occlusions in the MS arm of the trial compared to 46% (51/110) MCA occlusion in the DS-received MTE group. The rate of patients with tandem occlusions (combined occlusion of the cervical ICA and MCA) was higher in the DS-received MTE group [36% (40/110)] than in the MS group [19% (41/222)].

### Primary Endpoint Results

There was a significantly better primary outcome of functional independence (mRS 0–2) at 90 days in patients from the MS group compared to patients from the DS group [MS: 37% vs. DS-total: 26% (*p* = 0.024)/DS-received MTE: 24% (*p* = 0.017)]. Regression analysis showed a 1.7-fold increase in the good clinical outcome in MS-group patients (unadjusted OR 1,698; 95% KI 1,069–2,697; *p* = 0.025).

### Secondary Endpoint Results

#### Time Intervals

Time intervals between onset and first imaging were significantly shorter in DS patients than in the MS group [MS 89 min (IQR 67–116) vs. DS-total: 74 min (IQR 48–103)/DS-received MTE: 76 min (IQR 51–104)]. In patients who received IVT, rtPA was administered significantly earlier in the DS group than in MS patients [MS 120 min (IQR 89–155) vs. DS-total: 101 min (IQR 86–125)/DS-received MTE: 102 min (IQR 87–125)]. The time intervals changed in favor of MS patients with respect to the MTE. MS patients had their first angiographic run of the MTE procedure 114 min earlier than did patients from the DS-received MTE group [MS 180 min (IQR 152–218) vs. DS-received MTE: 294 min (IQR 98–397)]. Successful recanalization again occurred 112 min earlier in MS patients than in patients from the DS-received MTE group [MS 230 min (IQR 193–275) vs. DS-received MTE: 342 min (IQR 299–405)]. Relevant time intervals are listed in Table 1.

#### Transfer Times

**Figure 1:**
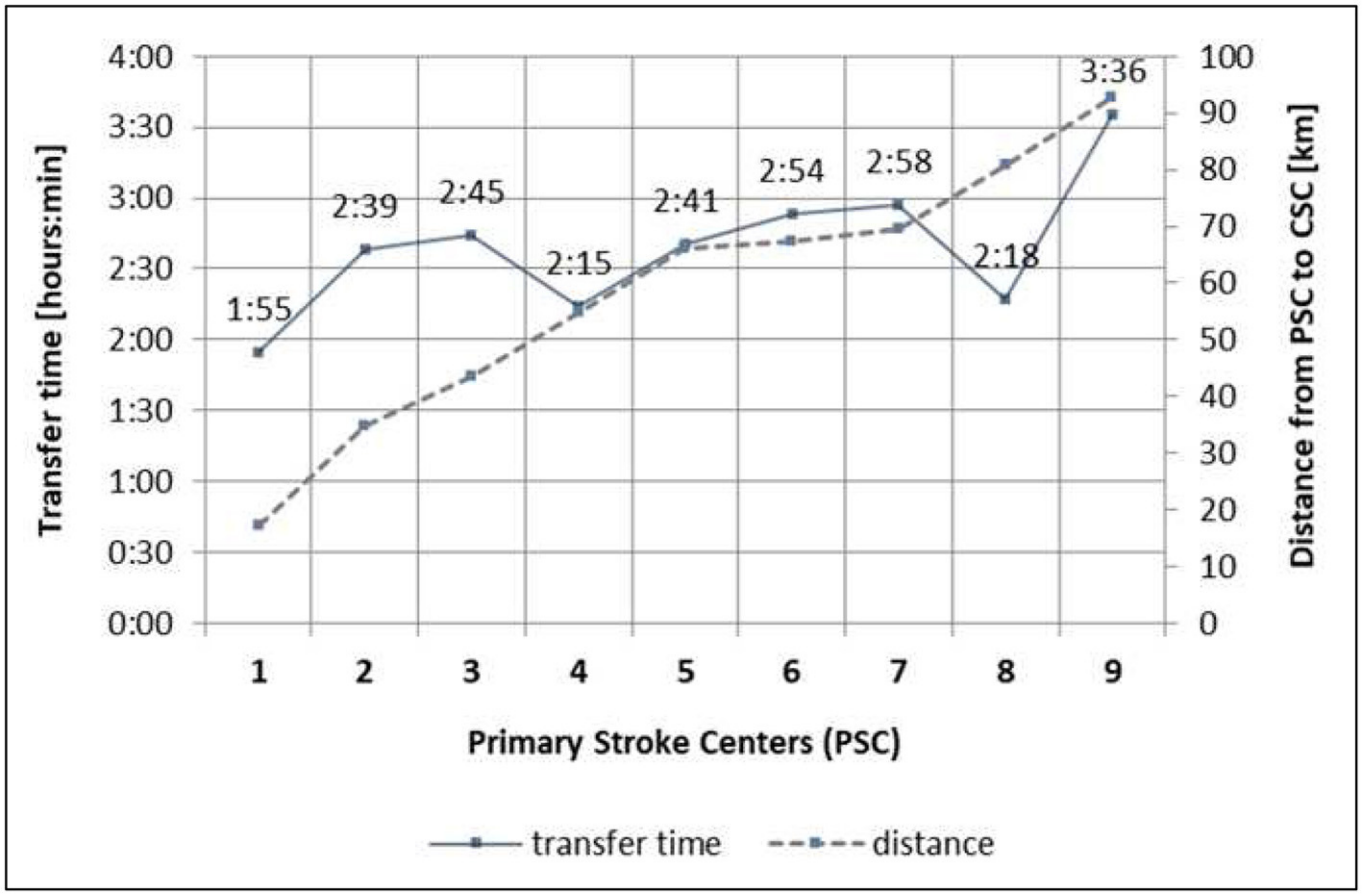
Neurovascular network with distances and transfer times from PSC to CSC. PSC, primary stroke centers; CSC, comprehensive stroke center (Freiburg); 1. PSC 1 with local stroke care, 2. PSC 2 with local stroke care, 3. PSC 3 with local stroke care, 4. PSC 4 with regional stroke care, 5. PSC 5 with regional stroke care, 6. PSC 6 with regional stroke care, 7. PSC 7 with regional stroke care, 8. PSC 8 with local stroke care, 9. PSC 9 with local stroke care.

Distances between the PSC and the CSC range from 17.1 to 96.4 km, determined by Google Maps. Individual patient transfer times were based on the emergency transport reports. Ninety-one (56%) patients from the DS group were transported via helicopter, 46 (28%) patients were transported by ambulance, and 26 patients were missing information concerning the mode of transport. Transfer times defined as time from first imaging at the PSC to arrival at the CSC ranged from 1 h 55 min to 3 h 36 min. Distances and mean transport times for the different PSCs are detailed in Figure 1.

#### Technical Efficacy Outcomes

In both groups, 99% of patients were treated with stent retrievers. The use of proximal balloon catheters did not differ (92% in both arms). There was no difference in the median number of stent retriever passes (one in both arms). Successful recanalization with angiographic demonstration of TICI scores of 2b (50–99% reperfusion) or 3 (complete reperfusion) was obtained in 159/220 (72%) of patients in the MS group and 80/110 (73%) of patients in the DS-received MTE group. Procedure-related information is summarized in Table 1.

#### Clinical Efficacy Outcomes

NIHSS at discharge differed significantly between the two arms of the study with a difference of five points on the NIHSS scale in favor of patients from the MS group [MS: 5 (range 2–14) vs. DS-received MTE: 10 (range 4–16)]. The mortality rates at 90 days did not differ significantly between groups (MS: 22% vs. DS-total: 22%/DS-received MTE: 17%).

A subgroup analysis of DS patients did not show a difference of good clinical outcome (mRS 0–2 vs. 3–6) when comparing patients with and without rtPA at PSC (*p* = 0.739). Good clinical outcome was not influenced by the different PSCs with differing transfer times in the DS group. In regression analysis, time to recanalization did show significant influence on good clinical outcome (*p* = 0.012). Forty-nine patients were recanalized outside the 6-h time window. Modified Rankin score from patients within 6-h time window was significantly better compared with that from patients outside the 6-h outcome (*p* = 0.042).

Symptomatic ICHs occurred more often in patients from the MS group [MS: 5% (10/220) vs. DS-received MTE: 2% (2/110)], such as asymptomatic lobar hematomas [MS: 5% (10/220) vs. DS-received MTE: 3% (3/110)] and subarachnoid hemorrhages [MS: 11% (24/220) vs. DS-received MTE: 8% (9/110)]. Those differences were statistically not significant. Clinical outcomes are summarized in Table 2.

**Table 2:** Clinical outcomes.

	**Mothership**	**Drip and**	**Drip and ship**	***p*-value**
		**ship total**	**received MTE**	
**Patients**	*N* = 222	*N* = 163	*N* = 110	
NIHSS at discharge, median (IQR)	5 (2–14)	11 (3–17)	10 (4–16)	**0.010**
mRS 0–2 at 90 days, *n* (%)	78 (37)	38 (26)	24 (24)	**0.017**
Mortality at 90 days, *n* (%)	46 (22)	33 (22)	17 (17)	0.306
Cerebral hemorrhage, *n* (%)	78 (38)	56 (39)	53 (52)	**0.023**
Subarachnoid hemorrhage, *n* (%)	24 (11)	9 (6)	9 (8)	0.585
Symptomatic ICH, *n* (%)	10 (5)	2 (1)	2 (2)	0.349
Asymptomatic ICH, *n* (%)	10 (5)	4 (3)	3 (3)	0.556

*Drip and ship-total, sent to the comprehensive stroke center (CSC) for mechanical thrombectomy (MTE); drip and ship-received MT, sent to CSC and received MTE; p-value, mothership vs. drip and ship-received MT; NIHSS, National Institutes of Health Stroke Scale; mRS, modified Rankin score; ICH, intracerebral hemorrhage. NIHSS at discharge unavailable for 5/385, mRS at 90 days unavailable for 24/385 patients, and neuroimaging 24 h after admission unavailable for 35/385. p-values that are significant are bold*.

## Discussion

In this study, functional independence (mRS ≤ 2) at 90 days was significantly less likely in DS patients than in the control group of patients treated under an MS paradigm (MS 37% vs. DS-total 26%, DS-received MTE 24%; *p* = 0.017).

A review on the recent literature comparing MS and DS paradigms shows contradictory findings. Six different trials including a total of 3,741 patients (MS: 1,428 and DS: 2,313) unanimously reported significantly shorter onset to recanalization times for the MS groups when compared to DS patients. Only in two of these trials was the reported clinical outcome [functional independence (mRS ≤ 2)] at 90 days was significantly better in MS patients than in DS patients (9, 10). In the remaining four trials (11–14), patients in the DS group fared better than MS patients without these differences reaching statistical significance (Table 3).

**Table 3:** Trials comparing drip-and-ship with mothership stroke care.

**Study**	**Study period**	***N***		**NIHSS**		***t*** **revasc**		**mRS 0–2 at 90 days**		***p-value***
		**MS**	**DS**	**MS**	**DS**	**MS**	**DS**	**MS**	**DS**	
Bücke (13)	2010–2015	124	817	16	15	260	348	31.5	37	0.709
Rinaldo (12)	2012–2016	62	78	17.8	18.5	277	420	61.7	66.2	0.580
Gerschenfeld (11)	2012–2016	59	100	NA	NA	198	248	50.8	61	0.26
Adams (14)	2014–2017	124	90	15	16.5	167	288	52	62	0.123
Weisenburger-Lile (9)	2012–2016	298	673	15.7	15.5	218	315	60.1	52.6	**0.018**
Froehler (10)	2014–2016	539	445	16.7	18	202	312	60	52	**0.02**
Taschner	2013–2016	222	110	15	15.5	230	342	37	24	**0.017**

*N, number of patients; NIHSS, National Institutes of Health Stroke Scale at baseline; t revasc, time from onset to revascularization/end of mechanical thrombectomy (MTE) procedure; mRS at 90 days, modified Rankin score at 90 days; MS, mothership paradigm; DS, drip-and-ship paradigm; NA, not available. p-values that are significant are bold*.

A recent meta-analysis by Ismael et al. came to the conclusion that patients who had an acute ischemic stroke admitted directly to a CSC may have better 90-day outcomes than those receiving DS treatment. Their analysis was based on eight studies including 2,068 patients who were selected. Patients who underwent MTE in an MS setting had better functional independence than those undergoing DS, whereas no differences were found between the treatment pathways in successful reperfusion, symptomatic intracranial hemorrhage, and 90-day mortality (15).

The contradictory results of the different trials might in part be explained by the fact that clinical outcomes in acute stroke patients depend on a large number of different factors including topography of a stroke network, local infrastructure, onset time to first medical response, traffic, weather, local door-to-needle times at PSCs, experience with thrombolysis at the PSC, different thresholds for MTE patient selection in LVO, distances and mode of transportation between PSC and CSC, door-to-groin times at the CSC, and experience of the operator at the CSC.

The findings of our trial are corroborating the results from Froehler and Weisenburger-Lile as well as the meta-analysis by Ismael, and they do not correlate with current guidelines, which recommend rapid transport of patients with a stroke to the nearest primary stroke capable of providing IVT therapy, even if they are being considered for MTE (AHA and ESO/ESMINT guidelines). The recently published RCT comparing endovascular thrombectomy with or without IVT in patients with LVO showed that MTE without prior IVT turned out to be non-inferior with regard to functional outcome, raising concerns that patients with LVO eligible for MTE are not adequately treated in a DS setting (3). There are even indications that IVT before MTE for LVO is associated with distal embolization, which in turn may reduce the chance that MTE can be attempted and recanalization can be achieved (16). But data on direct transfer of patients to CSC are still inconclusive as there are also trials which failed to prove non-inferiority of the MS model and proved its benefit prior ivtPA (17). Also, detection of stroke patients with and without LVO especially in atypical presentation remains challenging especially in the MS model; besides, sending all patients with suspected LVO directly to the CSC would substantially increase the patient volume in these centers (18).

Novel approaches to identify LVO patients in the pre-hospital phase are under scrutiny. Among these are stroke assessment tools applied by first-aid providers. Yet these tests already seem to have difficulties in reliably identifying ischemic stroke patients, let alone recognizing ischemic stroke patients with LVO. The current guidelines conclude that better stroke identification tools are needed in the pre-hospital setting (2). Corresponding research projects are currently getting under way.^2^ A different approach consists in piloting specialized stroke ambulances (mobile stroke units) that are supposed to speed up diagnosis, triage, and emergency treatment of patients with acute ischemic stroke symptoms (19, 20).

Our findings suggest that MTE for acute stroke treatment organized in a decentralized system could be of benefit for patients with LVO. Brekenfeld et al. have shown the benefits of shipping the neurointerventionalists to two PSCs, located at driving distances of 53 and 63 km from the CSC at the University Hospital Hamburg Eppendorf. This approach allows for paralleling necessary steps such as transportation, reevaluation, and angiography instead of a more time-consuming sequential organization of stroke patient care (21). This is especially crucial since our data in accordance with others show that time to recanalization has a significant influence on good clinical outcome. Thus, speeding up timelines and eliminating unnecessary and time-consuming steps of acute stroke care organization especially in patients with LVO at PSC are some of the challenging needs to be worked on. In CSCs with greater catchment areas, physicians could alternatively be trained at the PSC to perform MTE locally. Rinaldo et al. reasoned that it may be beneficial to expand the availability of endovascular revascularization services to lower-volume hospitals to minimize the morbidity associated with transfer to larger endovascular centers. In a study that included 8,533 patients from 118 institutions, they were able to demonstrate significantly lower mortality rates and mortality indices in high-volume centers (>132 MTE/year) compared to medium- and low-volume centers (22). This effect might not only be explained by more experienced neurointerventionalists in high-volume centers but also reflects the quality of the intensive care management elements of peri-interventional stroke management based on the current evidence, pathophysiologic considerations, and personal experience in a larger CSC (23).

### Study Limitations

Data for this monocentric trial were collected in a non-randomized fashion. The retrospective analysis of the prospectively maintained database included self-adjudicated primary imaging and clinical outcomes. We were confronted with the methodological problem on how to deal with patients diagnosed with LVO at the PSC secondarily sent to the CSC for MTE and who finally did not receive an MTE for different reasons (e.g., improvement under IV Alteplase and extended core infarct on secondary imaging at the CSC). Patients with LVO who improved prior to transfer from PSC to CSC were not included, leading to a potential time-reset effect (24). The findings of our trial reflect the specifics of our stroke network covering vast rural areas, and the generalizability of these results may thereby be limited.

### Conclusion

Our data suggest that patients who had an acute ischemic stroke admitted directly to a CSC may have better 90-day outcomes than those transferred secondarily for thrombectomy from a PSC. Also, in accordance with others, time to recanalization has an especially significant influence on good clinical outcome with the necessity of eliminating influenceable time-consuming steps in acute stroke organization. As a consequence, on the one hand, we have come up with a study of an acute LVO detected by LVO scoring in the field with helicopter-based direct transfer to the CSC^2^ as well as a reorganization of acute stroke care in DS patients bypassing secondary imaging at CSC with direct transfer to the angio-suite.

## Data Availability Statement

The original contributions presented in the study are included in the article/Supplementary Material, further inquiries can be directed to the corresponding author/s.

## Ethics Statement

The studies involving human participants were reviewed and approved by Ethics Committee, Faculty of Medicine, University Freiburg. Written informed consent for participation was not required for this study in accordance with the national legislation and the institutional requirements.

## Author Contributions

CT, AT, and W-DN: substantial contributions to the conception or design of the work. All authors: acquisition, analysis, or interpretation of data for the work, drafting the work or revising it critically for important intellectual content, final approval of the version to be published, and agreement to be accountable for all aspects of the work in ensuring that questions related to the accuracy or integrity of any part of the work are appropriately investigated and resolved.

## Conflict of Interest

The authors declare that the research was conducted in the absence of any commercial or financial relationships that could be construed as a potential conflict of interest.

## Publisher's Note

All claims expressed in this article are solely those of the authors and do not necessarily represent those of their affiliated organizations, or those of the publisher, the editors and the reviewers. Any product that may be evaluated in this article, or claim that may be made by its manufacturer, is not guaranteed or endorsed by the publisher.

## Footnotes

^1^https://en.wikipedia.org/wiki/Freiburg_(region) (accessed December 7, 2020).

^2^LESTOR trial: https://www.drks.de/drks_web/setLocale_EN.do.
