# Supplementary material for: Drip-and-Ship for Thrombectomy Treatment in Patients With Acute Ischemic Stroke Leads to Inferior Clinical Outcomes in a Stroke Network Covering Vast Rural Areas Compared to Direct Admission to a Comprehensive Stroke Center
Source: Front Neurol. 2021 Nov 1;12:743151. doi: 10.3389/fneur.2021.743151 (PMC8591070; doi:10.3389/fneur.2021.743151)
Supplement: Supplementary file 1 [file Table_1.DOCX]

Supplementary Material

**Online supplement 1. Reason for not performing mechanical thrombectomy in drip and ship patients**

| **Patients** | N=53 |
| --- | --- |
| Extended core infarct | 25 |
| No LVO | 12 |
| Clinical improvement after IVT | 8 |
| ICA occlusion, circle of Willis open | 5 |
| Extended time window >6h | 3 |

LVO= Large vessel occlusion; IVT= intravenous thrombolysis with alteplase; ICA= internal carotid artery
